# Integrated analysis of genes encoding ATP‐dependent chromatin remodellers identifies *CHD7* as a potential target for colorectal cancer therapy

**DOI:** 10.1002/ctm2.953

**Published:** 2022-07-05

**Authors:** Xingyan Zhang, Yaoyao Zhou, Zhenyu Shi, Zhenfeng Liu, Hao Chen, Xiaochen Wang, Yiming Cheng, Lishan Xi, Xuanyuan Li, Chunze Zhang, Li Bao, Chenghao Xuan

**Affiliations:** ^1^ The Province and Ministry Co‐sponsored Collaborative Innovation Center for Medical Epigenetics, Key Laboratory of Immune Microenvironment and Disease (Ministry of Education), Department of Biochemistry and Molecular Biology Tianjin Medical University Tianjin China; ^2^ Tianjin Medical University Cancer Institute and Hospital, National Clinical Research Center for Cancer, Key Laboratory of Cancer Prevention and Therapy, Tianjin's Clinical Research Center for Cancer, Key Laboratory of Breast Cancer Prevention and Therapy Tianjin Medical University, Ministry of Education Tianjin China; ^3^ Tianjin Institute of Coloproctology, Department of Colorectal Surgery Tianjin Union Medical Center Tianjin China

## Abstract

**Background:**

Genes participating in chromatin organization and regulation are frequently mutated or dysregulated in cancers. ATP‐dependent chromatin remodelers (ATPCRs) play a key role in organizing genomic DNA within chromatin, therefore regulating gene expression. The oncogenic role of ATPCRs and the mechanism involved remains unclear.

**Methods:**

We analyzed the genomic and transcriptional aberrations of the genes encoding ATPCRs in The Cancer Genome Atlas (TCGA) cohort. A series of cellular experiments and mouse tumor‐bearing experiments were conducted to reveal the regulatory function of CHD7 on the growth of colorectal cancer cells. RNA‐seq and ATAC‐seq approaches together with ChIP assays were performed to elucidate the downstream targets and the molecular mechanisms.

**Results:**

Our data showed that many ATPCRs represented a high frequency of somatic copy number alterations, widespread somatic mutations, remarkable expression abnormalities, and significant correlation with overall survival, suggesting several somatic driver candidates including chromodomain helicase DNA‐binding protein 7 (*CHD7*) in colorectal cancer. We experimentally demonstrated that CHD7 promotes the growth of colorectal cancer cells in vitro and in vivo. CHD7 can bind to the promoters of target genes to maintain chromatin accessibility and facilitate transcription. We found that CHD7 knockdown downregulates AK4 expression and activates AMPK phosphorylation, thereby promoting the phosphorylation and stability of p53 and leading to the inhibition of the colorectal cancer growth. Our muti‐omics analyses of ATPCRs across large‐scale cancer specimens identified potential therapeutic targets and our experimental studies revealed a novel CHD7‐AK4‐AMPK‐p53 axis that plays an oncogenic role in colorectal cancer.

## Graphical abstract

Graphical Abstract: (1) The genomic analyses of ATPCRs across large‐scale cancer specimens identified potential therapeutic targets, including CHD7 in colorectal cancer. (2) CHD7 promotes chromatin accessibility and gene transcription in colorectal cancer cells. (3) Our study reveals a novel CHD7‐AK4‐AMPK‐p53 axis that plays an oncogenic role in colorectal cancer.

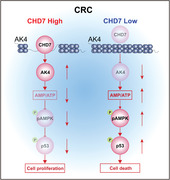

## 1 INTRODUCTION

Nucleosome, the basic functional unit of chromatin, consists of 146 base pairs of DNA wrapped around a histone octamer, which is assembled by two molecules of histones H2A, H2B, H3, and H4. Chromatin exists in two forms: (1) densely arranged heterochromatin that is inaccessible to transcriptional machinery and therefore contains inactive genes and (2) open and accessible euchromatin, encompassing active genes.^1^ Chromatin structural regulation is achieved through the collaboration of multiple regulatory pathways, including DNA modifications, chromatin remodelling, histone modifications, histone variants, and non‐coding RNAs.^2^ The dynamic and strictly controlled regulation of chromatin structures is very important for the precise establishment of genome‐wide epigenetic landscape and gene expression to regulate cell proliferation, cell differentiation, and organismic development.^1^, ^3^ ATP‐dependent chromatin remodellers (ATPCRs) utilize energy derived from hydrolysis of ATP to alter DNA–histone interactions within nucleosomes. These modulators can catalyse chromatin transformations, such as sliding the histone octamer across the DNA, altering the nucleosomal DNA conformation, and changing the histone octamer composition.^4^ ATP‐dependent remodelling is crucial for both the assembly and dissolution of chromatin structure.^5^ There are 37 genes encoding ATPCRs in mammals. Each of the identified ATPCRs contains an ATPase subunit belonging to the SNF2 (sucrose nonfermenting 2) superfamily. Based on other conserved domains, these ATPCRs are further classified as the SWI (mating type switching)/SNF, INO80 (inositol), ISWI (imitation switch), and CHD (chromodomain helicase DNA‐binding) families.^6^

Disruption of chromatin regulation can lead to detrimental results. The role of chromatin regulation in diseases has been extensively studied recently through exome‐ and genome‐wide sequencing, and mutations in genes participating in chromatin regulation and organization have been identified in more than 50% of cancers.^7^ Cancer is a disease derived from mutations in somatic cells. Gene copy number alteration (amplification and deletion), somatic mutation, and gene fusion are important genetic factors for persistent phenotypic changes. At the same time, epigenetic states are flexible yet hereditary through cell divisions and determine the cancer mutational landscape.^8^ Mutations that affect the epigenome are highly effective mechanisms to rearrange cellular circuitry because they can regulate a large number of target genes simultaneously. The extensively characterizing of epigenetic mutations by unbiased multi‐omics analysis will open a broad new field for basic and clinical discovery.

The role of chromatin remodelling in cancer development was initially indicated by the identification of biallelic deletions of *SNF5* in malignant rhabdoid tumours.^9^ Forty‐one per cent of renal cell cancer patients carry mutations in *BAF180* (one member of the ‘BAF’ SWI/SNF complex),^10^ whereas >50% of ovarian clear cell carcinomas harbour inactivating *ARID1A* mutations.^11^ *CHD1* deletions and inactivated mutations comprise founder events (together with Speckle‐type POZ protein mutations) in the ‘ETS‐negative’ subtype of prostate cancer,^12^ and *CHD4*, another ATPCR gene, has frequent deletion in endometrial cancers.^13^ In this study, by integrating multi‐omics data, we profiled the genomic aberrations and the transcriptional dysregulation of 37 ATPCR encoding genes in various cancer types across the cohort of The Cancer Genome Atlas (TCGA) programme and then investigated the role of CHD7 in colorectal cancer (CRC) in depth.

## 2 RESULTS

### 2.1 Genomic aberrations of ATPCRs in 32 types of cancer

**FIGURE 1:**
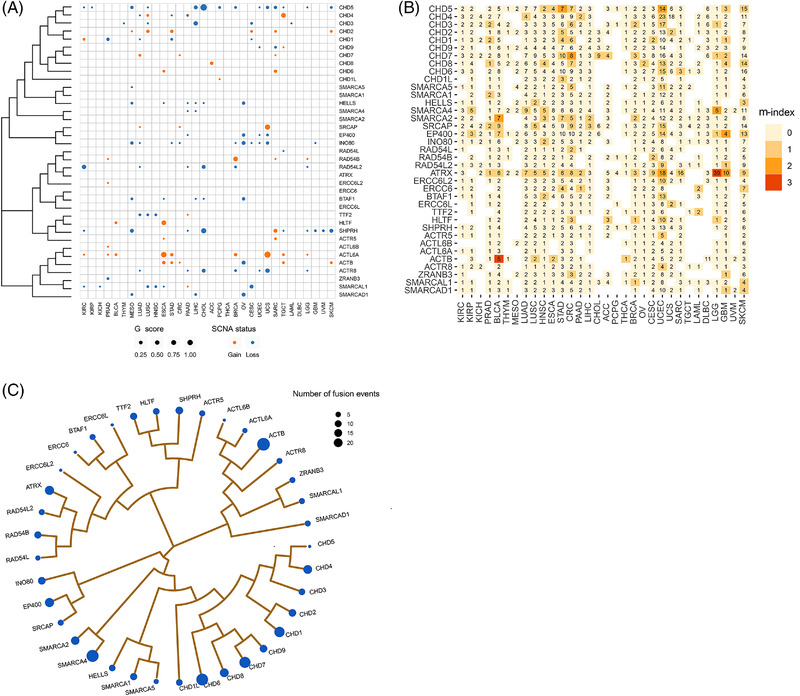
Genomic aberrations of ATP‐dependent chromatin remodellers (ATPCRs) across cancer types. (A) Bubble plot shows the *G*‐scores and somatic copy number alterations (SCNAs) status. The size of the bubble represents *G*‐score; colours denote SCNAs status. (B) Heat map shows the somatic mutation frequencies and *m*‐indexes of the ATPCRs. Cells are labelled with the mutation frequency (%) with gradient colours that denote *m*‐index. (C) Summary of the fusion event numbers of the ATPCRs. The area of the bubble represents the number of fusion events. The phylogenetic trees were generated by maximum likelihood methods based on protein sequences of ATPCRs

Somatic copy number alterations (SCNAs) affect a substantial part of genes in cancers and play a central role in cancer diagnostics and therapeutics.^14^ Single‐nucleotide polymorphism array profiles of 11 411 pan‐cancer specimens from TCGA were used to analyse SCNAs of ATPCRs (Table S1). We used GISTIC 2.0^15^ to identify deletion and amplification peaks and obtained the *G*‐score of each peak for each cancer type. After a filtering step based on *q*‐value and *G*‐score, we identified 141 significantly deleted or amplified peak regions across all cancer types, and 32 of 37 ATPCRs were located in peak regions of at least one cancer type (Table S2). Among them, seven ATPCR encoding genes were both recurrently amplified or deleted in different cancers, whereas the copy number changes of the other genes were consistent in different cancers, either gain or loss. In particular, *ACTL6A*, *CHD2*, and *ACTB* were identified with amplified events in at least five cancer types, whereas *CHD5*, *INO80*, *SHPRH*, *ACTR8*, *HELLS*, *RAD54L2*, *BTAF1*, *SMARCAL1* and *CHD3* were found with deleted events in at least five cancer types (Figure 1A and Table S3). Notably, most cancer types exhibited recurrent ATPCR‐associated SCNA events: pancreatic adenocarcinoma (*n* = 10), oesophageal carcinoma (*n* = 8), lung adenocarcinoma (*n* = 8), mesothelioma (*n* = 8), sarcoma (SARC, *n* = 8), and testicular germ cell tumours (*n* = 7). Only lymphoid neoplasm diffuse large B‐cell lymphoma and thyroid carcinoma had no ATPCR‐associated SCNAs (Figure 1A). These results demonstrated that ATPCRs had a wide range of SCNAs across most cancer types, suggesting that SCNAs of ATPCRs are not only attributable to genomic instability but may also have potential leading roles in tumour progression at a pan‐cancer level. We calculated the pan‐cancer *G*‐score for each gene using the unweighted summation of the *G*‐score of each cancer type. *ACTL6A*, *CHD2*, and *ACTB* had the highest pan‐cancer *G*‐score for amplification, whereas *CHD5*, *SHPRH*, *INO80*, and *ACTR8* had the highest pan‐cancer *G*‐score for deletions (Figure S1A and Table S3). Based on the aforementioned results, we identified 32 potential cancer‐causing ATPCRs driven by SCNAs in 32 cancer types.

Based on TCGA whole‐exome sequencing data, five complementary methods were used to characterize the somatic mutations (single base mutations and small indels) of ATPCRs (Figure 1B and Table S1). Eventually, seven ATPCR encoding genes, including *ATRX*, *CHD5*, *CHD7*, *SMARCA4*, *SMARCA2*, *EP400*, and *ACTB*, were identified as driver genes by at least two algorithms in certain cancer types (Figure 1B and Table S4). Among them, ATRX was frequently mutated in low‐grade gliomas (39%), uterine corpus endometrial carcinoma (UCEC, 18%), and glioblastoma multiforme (10%). *CHD5* was frequently mutated in stomach adenocarcinoma (7%) and UCEC (14%) (Figure 1B), whereas CHD7 mutations were identified in 8% of CRC cases (Figure 1B, Table S5). Although only seven ATPCR encoding genes exhibit a significant mutation rate, ten of the other ATPCRs had a considerable mutation frequency (>5%) in at least five cancer types, which may also be relevant to tumour development.

To identify the transcript fusion of ATPCRs at the pan‐cancer level, we obtained the fusion data of TCGA from the TumorFusions database^16^ (Table S2) and identified 195 fusion transcripts involving 37 ATPCRs in 30 of 32 cancer types (Table S6), which were relatively rare compared to SCNVs and mutations. We identified 15 recurrent fusion gene pairs among all cancer types, with *CHD7‐TOX* (*n* = 4), *EP400‐SFSWAP* (*n* = 4), and *SMARCA4‐DNM2* (*n* = 4) having the highest frequencies (Figure S1B and Table S6). Five ATPCR encoding genes, namely *CHD6*, *ACTB*, *SMARCA4*, *CHD1*, and *CHD7*, were associated with more than 10 fusion events (Figure 1C). Breast invasive carcinoma (45/1119) and SARC (19/263) had the highest frequency of ATPCR fusion events (Table S6). Although only 180 of 9950 (1.81%) tumour specimens showed fusion events of ATPCRs, transcript fusions were still regarded as remarkable genomic aberrations and may be associated with tumour progression, especially for those recurrently presented in multiple cancer types.

### 2.2 Expression and prognosis roles of ATPCRs across cancers

**FIGURE 2:**
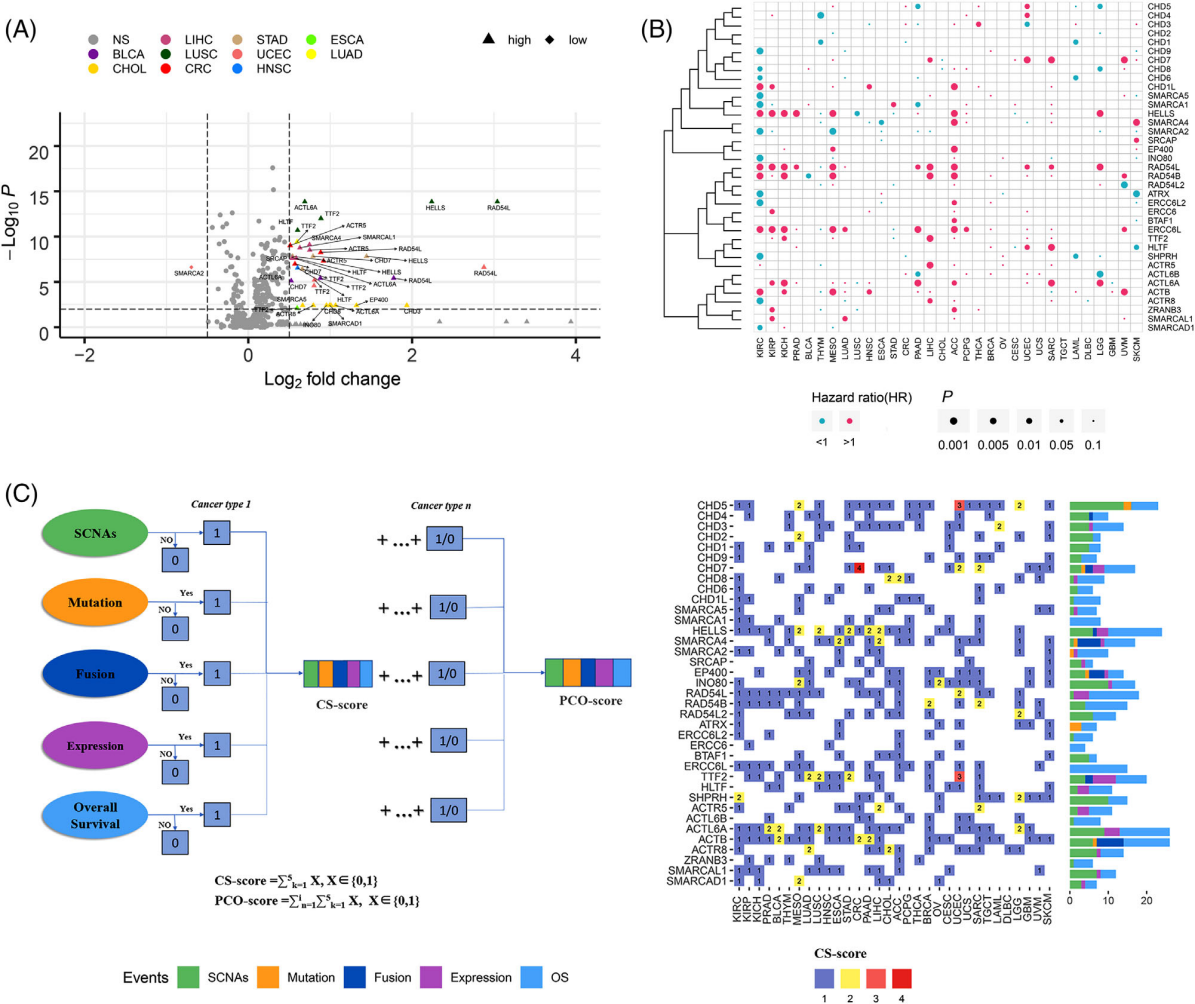
The mRNA expression, hazard ratios (HRs), and pan‐cancer overall (PCO) scores of ATP‐dependent chromatin remodeller (ATPCRs) across cancers. (A) The volcano plot indicates the differentially expressed ATPCRs between tumour and normal tissues: grey, not significantly differentially expressed genes; purple, significantly differentially expressed genes; triangles, genes in cancer with higher expression than normal tissues; and rhombus, genes in cancer with lower expression than normal tissues. (B) HRs of ATPCRs expression for the overall survival of each cancer type: the size of the bubble, *p*‐value of overall survival; the colour of the bubble, *P* < 0.1 and HR > 1, which represented poor prognostic (purple) and *P* < 0.1 and HR < 1, which represented good prognostic (blue). (C) The flow chart shows the workflow of calculating cancer‐specific (CS)‐ and PCO‐score based on genomic aberrations, transcriptional dysfunctions, and prognosis of patients related to ATPCRs. The right heat map indicates CS‐score, and the bars in flank display the PCO‐scores of each ATPCR. The colours of bar represent the components of PCO‐scores corresponding to different events, including somatic copy number alterations (SCNAs) (green), mutation (orange), fusion (blue), mRNA expression (purple), and overall survival (light blue)

We then analysed the transcriptional dysregulation of ATPCRs between tumours and adjacent tissues. An in‐depth analysis of the RNA sequencing (RNA‐seq) profiles of TCGA dataset (Table S1) showed that ATPCRs are generally highly expressed among coding genes (Figure S2A), and 34 of 37 ATPCRs had fragments per kilobase million (FPKM) of ≥1 in at least 90% of specimens from all cancer types, whereas only *CHD5*, *ERCC6*, and *RAD54B* did not meet this standard (Figure S2B and Table S7), indicating the broad expression of ATPCRs. Subsequently, differential expression analysis for 22 cancer types (because 10 cancer types lacked matched normal specimens data) versus adjacent normal specimens observed 18 differently expressed ATPCRs in 10 different cancer types. Seventeen of the 18 genes were upregulated in tumour specimens and only *SMARCA2* exhibited higher expression levels in the adjacent normal tissues in UCEC (Figure 2A). Especially, 7 of ATPCRs showed a significant overexpression in multiple cancer types, including *TTF2* (*n* = 6), *RAD54L* (*n* = 4), *ACTL6A* (*n* = 4), *CHD7* (*n* = 3), *HELLS* (*n* = 3), *HLTF* (*n* = 3), and *ACTR5* (*n* = 3) (Figure 2A and Table S8). Furthermore, we investigated the correlations between SCNAs and gene expression using a Pearson test. Using a *p*‐value threshold of .001, we found that the mRNA expression level of 32 ATPCR encoding genes was significantly correlated with their copy numbers, indicating the strong regulatory effect of SCNAs (Table S9, Figure S2C). In sum, these results demonstrated that 18 ATPCRs were differentially expressed between tumour and normal specimens, and their expression levels were significantly correlated with SCNAs.

To reveal the prognostic significance of ATPCRs, we performed survival analysis using the RNA expression level of ATPCRs as a univariate factor. Among the 32 cancer types, adrenocortical carcinoma, pheochromocytoma and paraganglioma, kidney chromophobe, prostate adenocarcinoma, and liver hepatocellular carcinoma typically had more poor‐prognostic‐associated ATPCR genes compared with other cancer types, whereas kidney renal clear cell carcinoma and thymoma showed more good‐prognostic‐associated ATPCRs (Figure 2B and Table S10). Among the 37 ATPCRs, we found that the high expression of four genes (*SMARCAL1*, *EP400*, *ERCC6*, and *CHD1L*) was associated with poor prognosis (hazard ratio [HR] > 1), whereas the high expression of the other four (*CHD6*, *CHD1*, *CHD2*, and *SMARCA2*) predicted a favourable prognosis (Figure 2B and Table S10). In particular, *ERCC6L* was the most significant prognostic gene whose expression level correlated with the overall survival (OS) of 15 different cancer types (Figure 2B and Table S11), suggesting that ATPCRs could be important prognostic factors.

### 2.3 Quantitative evaluation of driver potential of ATPCRs

To evaluate the cancer driver potential of ATPCRs, we determined a cancer‐specific score (CS‐score) for each ATPCR in unique cancer types by integrating five factors (recurrent SCNAs, significant mutations, recurrent fusions, different expression, and significant prognostic correlation) and then summed the CS‐scores of each cancer type to obtain a pan‐cancer overall score (PCO‐score) (Figure 2C). Among the 37 ATPCR encoding genes, *ACTB*, *ACTL6A*, *HELLS*, and *CHD5* had the highest PCO‐scores (>20) (Figure 2C). However, different genes showed significant PCO‐scores favouring different factors. For example, *CHD5*, *INO80*, and *SHPRH* were predominantly aberrated by SCNAs, whereas *ERCC6L* and *HELLS* were mostly related to survival relevance (Figure 2C and Table S11). When focusing on the CS‐scores of ATPCRs in individual cancer types, we observed that the maximum CS‐score corresponded to *CHD7* in CRC (CS‐score = 4) (Figure 2C and Table S12). In CRC, *CHD7* was amplified in 331 cases (53.7%) (Figure S3), mutated with a frequency of 8%, identified with four gene fusion events, and more overexpressed in tumour tissues than in the adjacent normal tissues. Moreover, alterations of *CHD7* were not only specific to CRC. *CHD7* also showed an increased copy number and an overexpression in 13 and 9 cancer types, respectively, suggesting its oncogenic roles in multiple types of cancer. Previous research on *CHD7* has focused on CHARGE syndrome and embryonic development, and little is known about its role in tumourigenesis. Therefore, we aimed to study the function of *CHD7* in CRC.

### 2.4 CHD7 promotes colorectal cancer cells growth in vitro and in vivo

**FIGURE 3:**
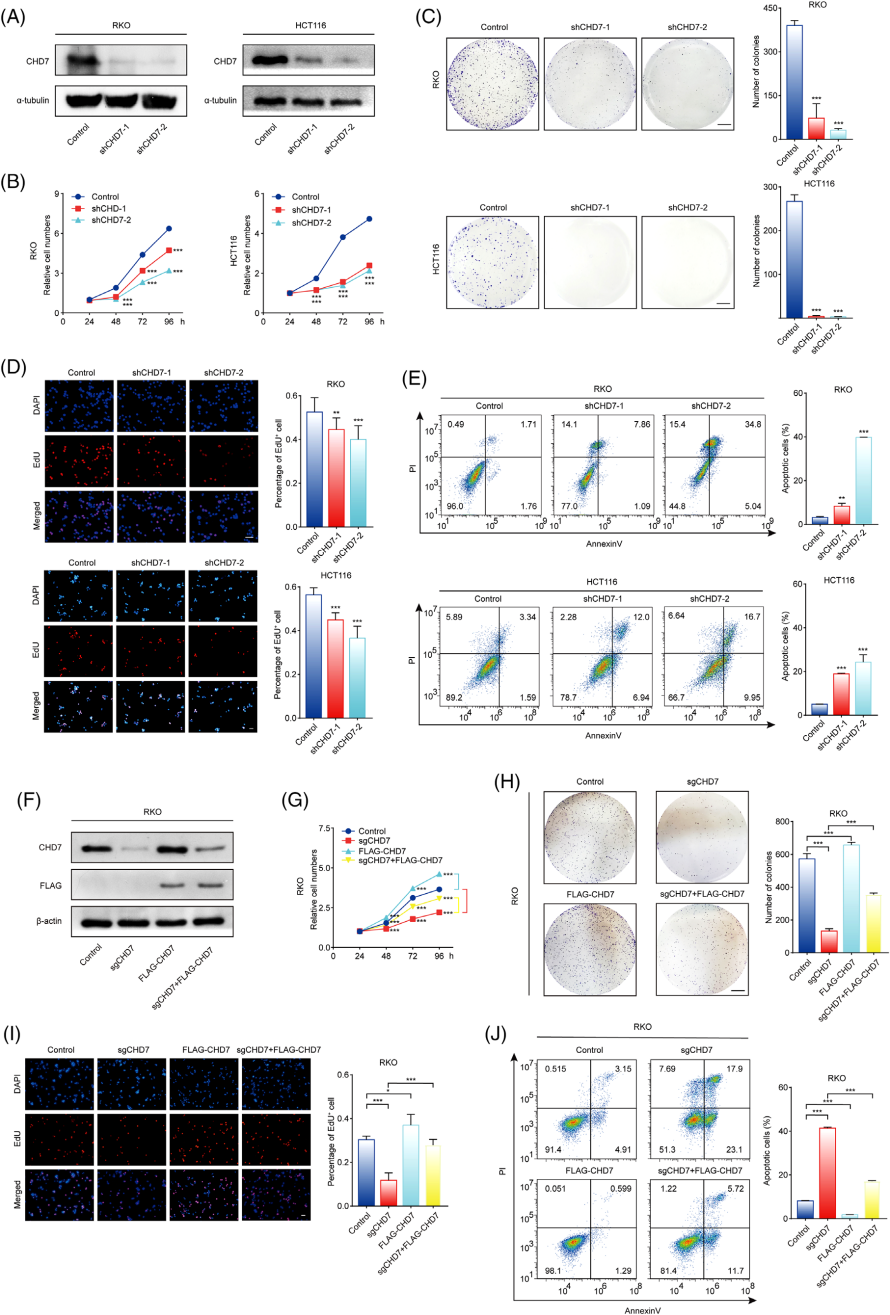
Chromodomain helicase DNA‐binding protein 7 (CHD7) promotes the growth of colorectal cancer cells in vitro. (A) CHD7 was knocked down by two independent short hairpin RNAs (shRNAs) in RKO and HCT116 cells. Knockdown efficiency was detected by Western blotting. (B) Depletion of CHD7 decreases the viability of colorectal cancer cells. Cell viability was measured using MTT assays. (C) RKO and HCT116 cells expressing that indicated shRNAs were maintained in culture media for 2 weeks and stained with crystal violet, and the number of colonies was counted. Scale bar, 5 mm. (D) CHD7 knockdown decreases the proliferation of colorectal cancer cells. RKO and HCT116 cells expressing control or *CHD7* shRNAs were subjected to EdU‐incorporation assays, and the percentage of EdU‐positive cells were calculated. Scale bar, 50 μm. (E) Depletion of CHD7 leads to cell apoptosis. CHD7‐depleted or control cells were stained with FITC‐labelled annexin V and propidium iodide, followed by apoptotic analysis using flow cytometry. (F) Western blot analysis of the expression of CHD7, FLAG‐CHD7, and β‐actin in cells stably expressing Cas9 together with control or *CHD7* sgRNAs, and transiently expressing vector or FLAG‐CHD7. (G) MTT assays showing the viability of indicated cells. (H) Colony formation assays were performed in indicated cells. Scale bar, 5 mm. (I) Results of EdU‐incorporation assays. Scale bar, 50 μm. (J) Cell apoptosis was detected by flow cytometry. For parts B–E and G–J, data are mean ± SD for *n* = 3; *P < 0.05, **P < 0.01, ***P < 0.001 (Student's *t* test)

To investigate the oncogenic role of CHD7 in CRC, we knocked down CHD7 expression in two CRC cell lines RKO and HCT116 using short hairpin RNAs (shRNAs), and the effective depletion of CHD7 was confirmed by Western blotting assays (Figure 3A). CHD7 knockdown substantially inhibited cell viability, as determined by MTT and colony formation assays (Figure 3B,C). Notably, CHD7 depletion not only suppressed proliferation, as tested by EdU incorporation assays (Figure 3D), but also triggered apoptosis, as determined by flow cytometry analysis of annexin‐V/propidium iodide‐stained RKO and HCT116 cells (Figure 3E).

FLAG‐CHD7 was then overexpressed in RKO cells (Figure 3F), and our results showed that, compared with control RKO cells, FLAG‐CHD7 overexpressed RKO cells grew faster in vitro, because the cell viability (Figure 3G), number of colonies (Figure 3H), and proportion of EdU‐positive cells (Figure 3I) increased, and the percentage of apoptotic cells decreased (Figure 3J). After we overexpressed FLAG‐CHD7 in *CHD7* knockout cells (RKO cells stably expressing *CHD7* sgRNA and Cas9) (Figure 3F), the inhibition of cell growth and the increase of apoptosis caused by CHD7 knockout were rescued (Figure 3G–J), indicating that the cell proliferation inhibition and the increase of apoptosis of CHD7 knockdown cells were indeed caused by the depletion of CHD7 proteins.

**FIGURE 4:**
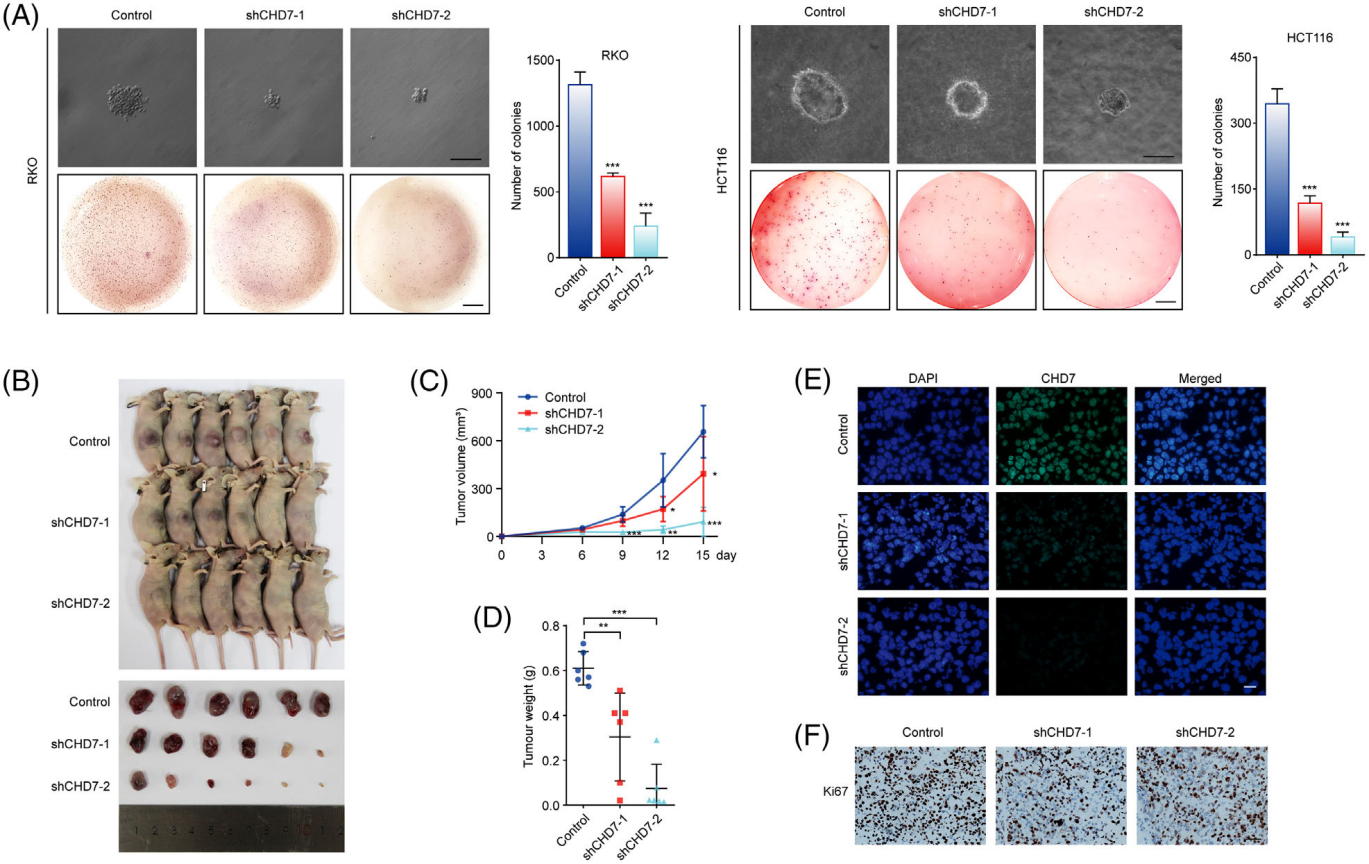
Chromodomain helicase DNA‐binding protein 7 (CHD7) knockdown inhibits colorectal tumour growth in vivo. (A) CHD7 knockdown decreased anchorage‐independent growth of colorectal cells. RKO and HCT116 cells expressing control or *CHD7* short hairpin RNAs (shRNAs) were loaded on soft agar. Representative images show a single colony (upper figures, scale bar, 100 μm) and the entire well (lower figures, scale bar, 5 mm). Data are mean ± SD for *n* = 3; ***P < 0.001 versus control (Student's *t* test). (B) CHD7 knockdown inhibits colorectal tumour growth in vivo. RKO cells expressing control or *CHD7* shRNAs were transplanted into female athymic nude mice. Tumours were stripped out 15 days later and photographed. (C) After implantation into mice, tumours were measured every 3 days using a Vernier calliper, and the volume was calculated according to the formula: *V* = *π*/6 × length × width^2^. Each bar represents the mean ± SD for six animal measurements, *P < 0.05, **P < 0.01, ***P < 0.001 versus control (Student's *t* test). (D) The tumours were weighed. Each bar represents the mean ± SD for *n* = 6, **P < 0.01, ***P < 0.001 versus control (Student's *t* test). (E) The tumours were stripped out from mice, and the frozen sections were stained with anti‐CHD7. 4‐6‐Diamidino‐2‐phenylindole (DAPI) staining was included to visualize the nuclei. Scale bar, 20 μm. (F) Immunohistochemical staining of the frozen sections using antibodies against Ki67. Scale bar, 50 μm

Furthermore, we used CHD7‐depleted RKO and HCT116 cells to perform colony formation experiments on soft agar, which can detect the ability of tumour cells to form colonies in a non‐adherent state and reflect the malignant proliferation ability of tumour cells. The results showed that *CHD7* knockdown significantly suppressed anchorage‐independent growth of CRC cells (Figure 4A).

To further analyse the effect of CHD7 knockdown on the development of CRC in vivo, we constructed a mouse tumour‐bearing model, which can stimulate the growth of tumour in vivo. RKO cells expressing control shRNAs or *CHD7* shRNAs were transplanted subcutaneously into nude mice (BALB/c, *n* = 6 for each group). The growth of the implanted tumours was measured over 15 days. The results indicated that the growth of tumours was significantly inhibited in athymic mice that had received tumours with CHD7 knockdown, as the tumour volume and weight were evidently inhibited in CHD7‐depleted groups (Figure 4B–D). The knockdown of CHD7 expression in the xenograft was confirmed by immunofluorescent staining of CHD7 in frozen sections of the tumours from mice (Figure 4E). Moreover, the malignancy of the tumour with CHD7 knockdown was much lower than that of tumours formed by control RKO cells as indicated by Ki‐67 staining (Figure 4F). Taken together, our data demonstrated the oncogenic role of CHD7 in CRC in vitro and in vivo.

### 2.5 Depletion of CHD7 activates the p53 signalling pathway

**FIGURE 5:**
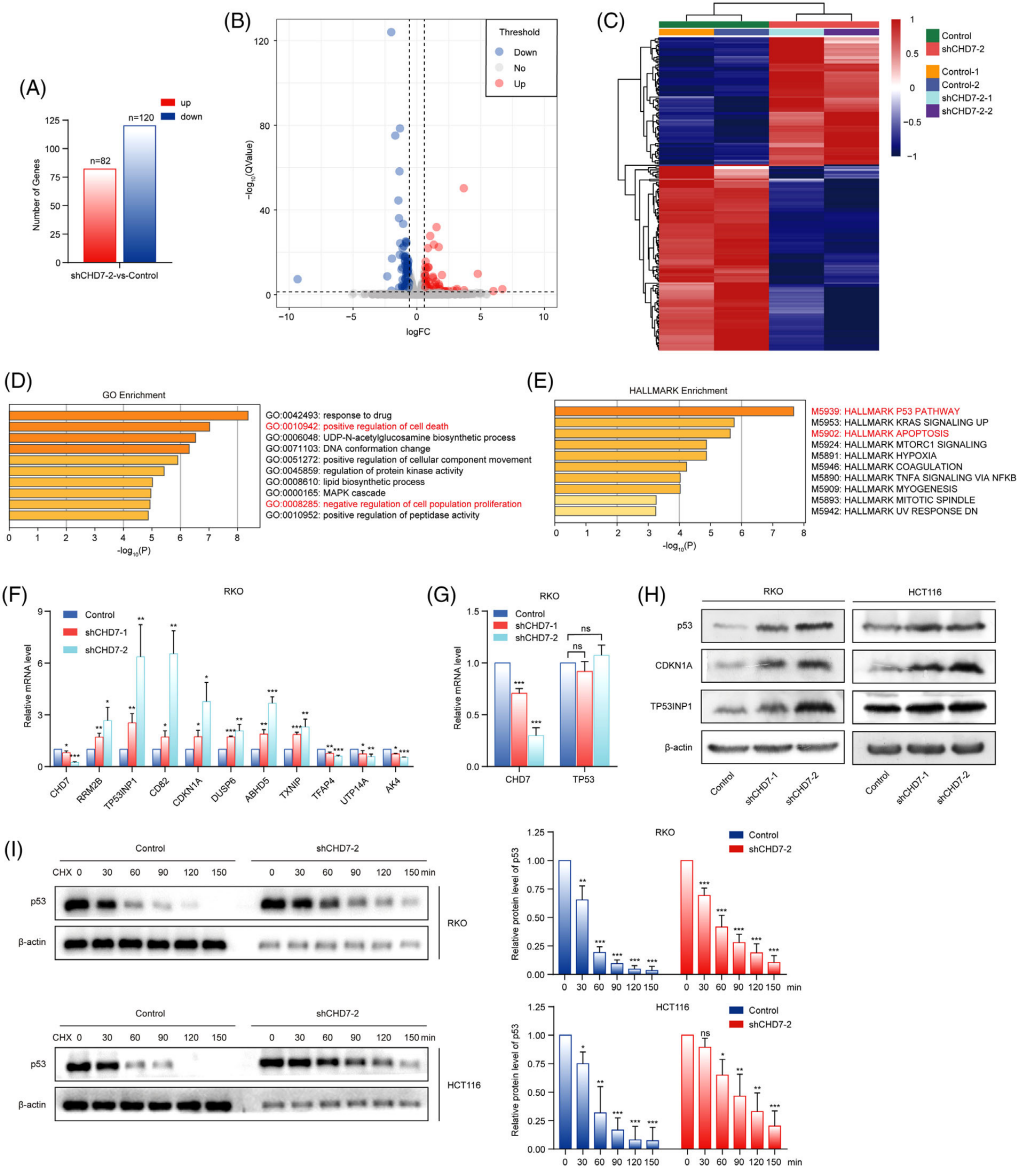
Depletion of chromodomain helicase DNA‐binding protein 7 (CHD7) activates the p53 signalling pathway. (A) The mRNAs from cells expressing control short hairpin RNAs (shRNA) or *CHD7* shRNA‐2 were extracted and subjected to RNA sequencing (RNA‐seq). At the significance of *q* < 0.05 and with foldchange ≥1.5, the number of dysregulated genes are shown. (B) Volcano plot shows the differentially expressed genes between RKO cells expressing control shRNAs and *CHD7* shRNA‐2. At the significance of *q* < 0.05 and with foldchange ≥1.5, significantly upregulated genes are shown as red dots, and downregulated as blue dots. (C) Heat maps show the expression of significantly dysregulated genes. (D) Gene ontology (GO) enrichment analyses of significantly dysregulated genes in CHD7 knockdown RKO cells. (E) Hallmark enrichment analyses of dysregulated genes in CHD7 knockdown RKO cells. (F) Total mRNA from RKO cells expressing indicated shRNAs was extracted and quantitative real‐time RT‐PCR assays were performed. Each bar represents the mean ± SD for *n* = 3, *P < 0.05, **P < 0.01, ***P < 0.001 versus control (Student's *t* test). (G) Quantitative real‐time RT‐PCR assays were performed using primers targeting p53. Each bar represents the mean ± SD for *n* = 3, ***P < 0.001 versus control (Student's *t* test). (H) Western blot analysis of the expression of p53, CDKN1A, TP53BP1, and β‐actin in RKO and HCT116 cells expressing control or *CHD7* shRNAs. (I) RKO and HCT116 cells expressing control shRNA or *CHD7* shRNA‐2 were treated with cycloheximide (CHX) (50 mg/ml) for 0, 30, 60, 90, 120, and 150 min, and then cell lysates were subjected to Western blotting

To decipher how CHD7 knockdown affects the growth of CRC cells, we applied an unbiased genomic approach to clarify the transcriptional programme regulated by CHD7 in RKO cells. RNA‐seq was performed to identify differentially expressed transcripts by CHD7 knockdown in RKO cells. Bioinformatic analyses revealed that, at the significance of *q* < .05, 120 genes were downregulated and 82 genes were upregulated in CHD7 knockdown cells with foldchange ≥1.5 (Figure 5A–C). Importantly, gene ontology (GO) term analysis of genes dysregulated upon CHD7 knockdown demonstrated a positive regulation of apoptotic process and a negative regulation of cell proliferation as the most enriched biological processes (Figure 5D). Furthermore, hallmark enrichment analyses identified the p53 signalling pathway as the most enriched pathway regulated by CHD7 knockdown (Figure 5E). The expression alteration of several representative genes associated with the p53 pathway was validated in control and CHD7‐depleted RKO cells by real‐time RT‐PCR assays. The mRNA expression levels of p53 target genes, such as *CDKN1A*, *RRM2B*, *TP53INP1*, *CD82*, and *DUSP6*, were upregulated following CHD7 knockdown (Figure 5F). Although no significant changes in p53 mRNA levels were detected in our RNA‐seq and real‐time RT‐PCR assays (Figure 5G), the protein levels of p53 and its targets, *CDKN1A* and *TP53INP1*, were significantly increased in CHD7‐depleted cells (Figure 5H), suggesting that CHD7 knockdown in CRC cell lines led to an increase in the protein stability of p53. To test this possibility, we treated control or CHD7‐knockdown RKO and HCT116 cells with cycloheximide, and the protein levels of p53 were examined. The results showed that CHD7 knockdown increased the stability of p53 protein (Figure 5I). In addition, CHD7 depletion (Figure S4A,C) did not affect the growth of p53‐mutant CRC cells (SW480) (Figure S4B) and HCT116 p53^−/−^ cells (Figure S4D), suggesting the importance of p53 in CHD7‐regulated cell growth. Then, we analysed the percentage of late‐stage and early‐stage cases in mutant *CHD7* and wild‐type *CHD7* groups of CRC patients with wild‐type p53. The analyses showed that among the wild‐type *TP53* TCGA CRC cases, the patients with wild‐type *CHD7* had a significantly higher percentage of late‐stage compared to cases with mutant *CHD7*, suggesting the carcinogenic potential of overexpressed wild‐type *CHD7* in p53 wild‐type CRC (Figure S4E). The RNA‐seq assays also revealed that the transcriptions of several genes, such as *UTP14A*, *TFAP4*, and*AK4*, which may function in the upstream of p53 protein to regulate its stability, were decreased by CHD7 depletion (Figure 5F), suggesting a potential mechanism underlying CHD7‐regulated p53 protein stability.

### 2.6 CHD7 knockdown decreases the promoter chromatin accessibility of target genes

**FIGURE 6:**
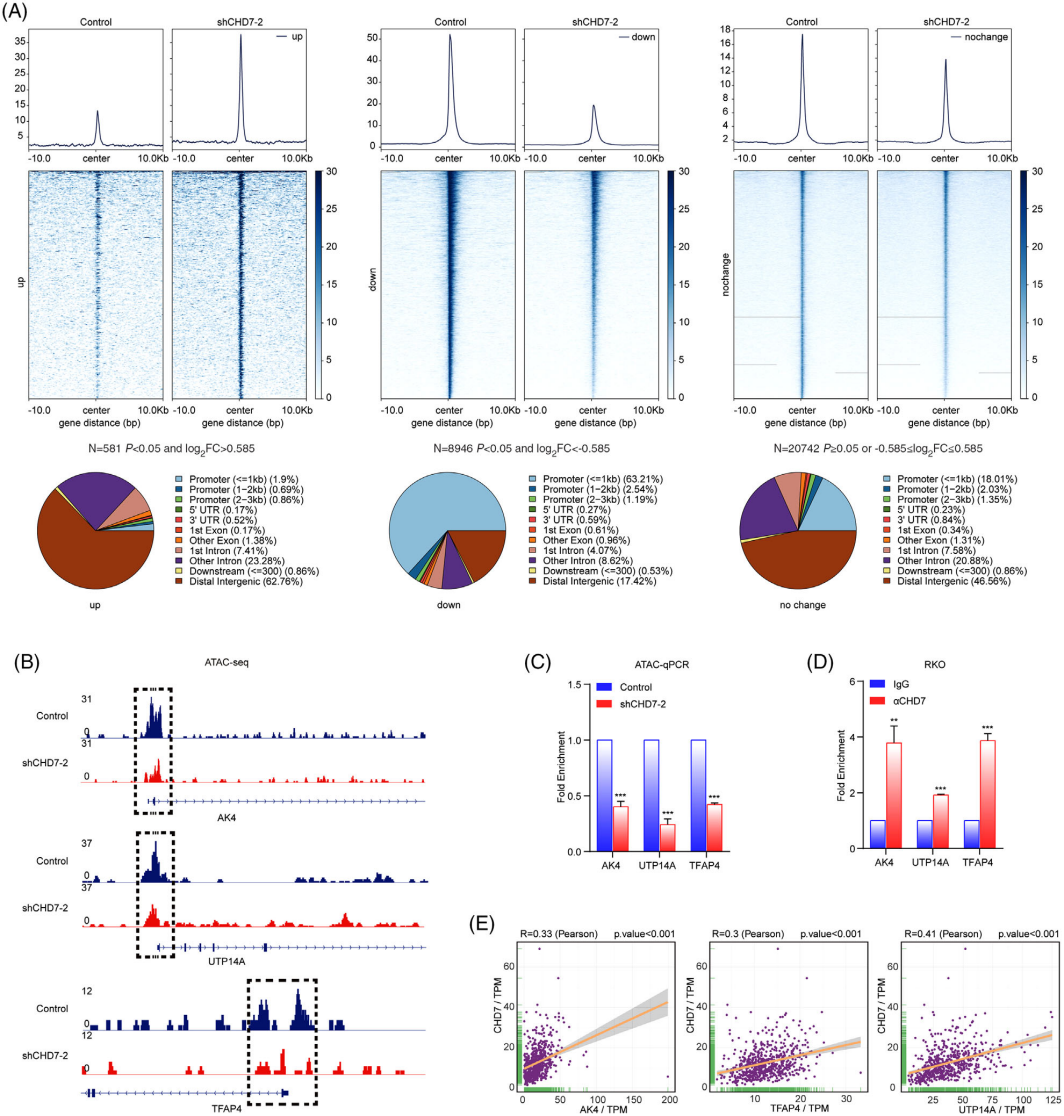
Chromodomain helicase DNA‐binding protein 7 (CHD7) knockdown decreases the chromatin accessibility of target genes. (A) ATAC‐seq was performed in CHD7 depleted RKO cells and control cells. At the significance of *p* < 0.05, and according to the foldchange of chromatin accessibility peak signal between control and CHD7 knockdown group, peaks were classified into three clusters. The profile diagrams and heat maps show chromatin accessibility levels in RKO cells expressing control or *CHD7* short hairpin RNAs (shRNAs), and the pie charts show the distribution of peaks in the three clusters across the genome. (B) The ATAC‐seq peaks on the promoter of target genes were visualized by Integrative Genomics Viewer (IGV). (C) ATAC‐qPCR assays and (D) ChIP assays were performed in RKO cells using primers targeting the promoters of *AK4*, *UTP14A*, or *TRAP4*. Each bar represents the mean ± SD for *n* = 3, **P < 0.01, ***P < 0.001 versus control (Student's *t* test). (E) Spearman correlation between *CHD7* and *AK4*, *UTP14A*, or *TFAP4* mRNA expression levels in The Cancer Genome Atlas (TCGA) COAD and READ specimens. *R*: Pearson's correlation coefficient

To further explore the role of CHD7 in transcriptional regulation as a chromatin remodeller, we performed ATAC‐seq in CHD7‐depleted RKO cells and control cells to detect the alteration of chromatin accessibility. Loss of CHD7 dramatically altered overall chromatin accessibility, resulting in increased accessibility at 581 sites and decreased accessibility at 8946 sites with *q* < 0.05 and foldchange ≥1.5 (Figure 6A). Peaks were clustered according to the change in accessibility caused by CHD7 knockdown. Cluster 1 exhibited increased accessibility upon loss of CHD7, and cluster 2 showed decreased accessibility. The decreased accessibility sites were primarily distributed in the promoter region across the genome (Figure 6A), suggesting that CHD7 knockdown downregulated gene transcription through decreasing chromatin accessibility in the promoters. The ATAC‐seq results were visualized by Integrative Genomics Viewer (Figure 6B), showing that the promoter chromatin accessibility of *UTP14A*, *TFAP4*, and*AK4* was decreased in CHD7‐depleted cells. This is consistent with the RNA‐seq results that the transcription of these genes was downregulated by CHD7 knockdown. Additional ATAC‐qPCR and ChIP assays demonstrated that CHD7 bound to the promoter region of these target genes to maintain chromatin accessibility (Figure 6C,D). Furthermore, the mRNA levels of *CHD7* and *AK4*, *UTP14A*, or *TFAP4* in TCGA CRC specimens were highly correlated (Figure 6E). Taken together, our data demonstrated that CHD7 binds to the promoter of target genes to maintain chromatin accessibility and facilitate gene transcription.

### 2.7 CHD7 knockdown increases the stability and activity of p53 through AK4‐AMPK‐p53 axis

**FIGURE 7:**
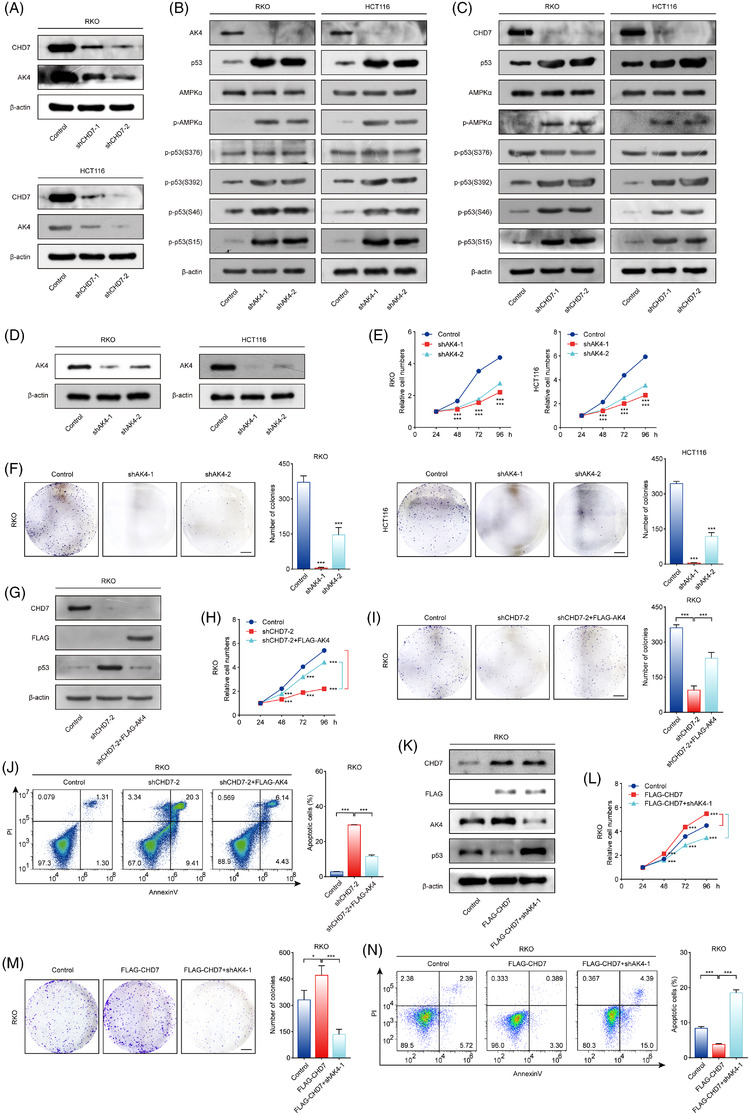
Chromodomain helicase DNA‐binding protein 7 (CHD7) knockdown increases the stability and activity of p53 through AK4‐AMPK‐p53 axis. (A) CHD7 knockdown decreases AK4 protein levels. The cell lysates from RKO and HCT116 cells expressing control or *CHD7* short hairpin RNAs (shRNAs) were subjected to Western blotting using indicated antibodies. (B) AK4 knockdown activated AMPK and increased the phosphorylation and stability of p53 protein. (C) CHD7 depletion increased the phosphorylation of AMPK and p53 protein. (D) Knockdown efficiency of shAK4 was detected by Western blotting. (E) Depletion of AK4 decreases the viability of colorectal cancer cells. Cell viability was measured using MTT assays. (F) RKO and HCT116 cells expressing control or *AK4* shRNAs were maintained in culture media for 2 weeks and stained with crystal violet, and the number of colonies was counted. Scale bar, 5 mm. (G) Western blot analysis of the expression of CHD7, FLAG‐AK4, p53, and β‐actin in indicated cells. (H) The viability of indicated cells was measured using MTT assays. (I) Colony formation assays were performed in indicated cells. Scale bar, 5 mm. (J) Cell apoptosis was detected by flow cytometry. For parts (H)–(J), data are mean ± SD for *n* = 3; *P < 0.05, **P < 0.01, ***P < 0.001 (Student's *t* test). (K) Western blot analysis of the expression of CHD7, FLAG‐CHD7, AK4, p53, and β‐actin in indicated cells. (L) The viability of indicated cells was measured using MTT assays. (M) Colony formation assays were performed in indicated cells. Scale bar, 5 mm. (N) Cell apoptosis was detected by flow cytometry. For parts (H)–(J) and (L)–(N), data are mean ± SD for *n* = 3; *P < 0.05, **P < 0.01, ***P < 0.001 (Student's *t* test)

AK4, an adenylate kinase, regulates the balance of intracellular ATP, ADP, and AMP levels.^17^, ^18^ We proposed that CHD7 may affect the protein stability of p53 through the transcriptional regulation of AK4. To test this possibility, we first detected the protein level of AK4 after CHD7 knockdown and found that the protein level of AK4 decreased considerably in response to CHD7 depletion (Figure 7A). It has been reported that AK4 knockdown led to an increase in cellular AMP/ATP ratio, resulting in the increase of AMP‐activated protein (AMPK) phosphorylation in multiple cell lines.^19^ We then knocked down the expression of AK4 and CHD7, respectively, and detected the phosphorylation of AMPK in RKO and HCT116 cells. The results showed that the level of AMPKα1‐T183/AMPKα2‐T172 increased upon AK4 and CHD7 knockdown (Figure 7B,C). AMPK is a regulator of cellular energy and metabolism, which can implement a metabolic checkpoint partially dependent on p53. After AMPK activation, p53 protein accumulates and activates the transcription of downstream genes involved in cell cycle arrest and apoptosis.^20^, ^21^ Therefore, we proceeded to detect the phosphorylation of p53 in AK4 or CHD7 knockdown cells, and the results revealed that the phosphorylation of p53 at S392, S15, and S46 increased in response to AK4 and CHD7 depletion (Figure 7B,C). The phosphorylation of p53 at S15 was reported to enhance the protein stability of p53,^22^ and the phosphorylation at S46 and S392 promotes the transcriptional activity of p53, leading to p53‐mediated cell cycle arrest and apoptosis.^23^, ^24^, ^25^ This is consistent with our previous results that CHD7 depletion increased the expression of p53 and its target genes (Figure 5H). In conclusion, we found that CHD7 knockdown leads to decreased transcription of AK4 and subsequently increased AMPK and p53 phosphorylation, resulting in increased protein stability and transcriptional activity of p53.

To further evaluate whether AK4 regulates the growth of CRC cells, MTT assays and colony formation assays were performed in control or AK4 knockdown RKO and HCT116 cells (Figure 7D). The results showed that the growth of these two CRC cell lines was inhibited by AK4 knockdown (Figure 7E,F). To prove that the inhibition of cell proliferation, increase in apoptosis, and enhancement of p53 stability caused by CHD7 knockdown were mediated by the downregulation of AK4, we transfected FLAG‐AK4 expression constructs into CHD7 knockdown cells (Figure 7G), and p53 protein levels, as well as cell growth and apoptosis, were detected. The results showed that AK4 overexpression rescued the phenotype induced by CHD7 depletion: the p53 protein stability declined (Figure 7G); the cell viability increased (Figure 7H,I); and the percentage of apoptotic cells decreased (Figure 7J). When AK4 was knocked down by its specific shRNA in cells with CHD7 overexpressed, AK4 depletion inhibited the phenotype caused by CHD7 overexpression: the p53 protein stability increased (Figure 7K); the cell growth decreased (Figure 7L,M); and the percentage of apoptotic cells increased (Figure 7N). Taken together, our data demonstrated that CHD7 promotes the growth of CRC through the AK4‐AMPK‐p53 axis.

## 3 DISCUSSION

Cancer develops through cumulative disruptions of the controls of cell proliferation, cell death, immortality, angiogenesis, cell invasion, and metastasis. This evolutionary process needs the stable maintenance of new malignant traits to accumulate in clonal lineages. Epigenetic states are flexible and hereditary through multiple cell divisions. Disruption in chromatin regulation can have serious consequences. Advances in genome‐wide sequencing technology have uncovered the prevalent mutations in genes encoding epigenetic factors. In some types of cancers, such mutations are the only genetic abnormality, evidencing their initiating and pathogenic function, rather than permitting passenger mutations.

Chromatin remodelling is a highly coordinated process that regulates gene expression through cooperating with transcriptional machinery. ATPCRs play a key role in organizing genomic DNA within chromatin. In this study, by integrating multi‐omic profiles, we systematically analysed the genomic and expressional aberrations of 37 ATPCRs encoding genes across the entire TCGA data cohort. Our analyses showed that many ATPCRs represented a high frequency of SCNAs, widespread somatic mutations, remarkable expression abnormalities, and significant correlation with OS, suggesting that ATPCRs may contribute to carcinogenesis in a variety of aspects. In recent years, some small molecular inhibitors targeting epigenetic factors have achieved good therapeutic effects in preclinical experiments. Therefore, our analyses are of great value in providing potential clinical therapeutic targets.

ATPCRs are classified into the SWI/SNF, ISWI, INO80, and CHD families. The CHD family consists of nine members, CHD1–9, which all have chromatin organization modifier (CHROMO) domains that bind modified histones specifically, and an SNF2‐like ATP‐dependent helicase domain to promote nucleosome mobilization.^6^ *CHD7* haploinsufficiency is a major cause of CHARGE syndrome, a genetic disorder occurring with a prevalence of approximately 1 in 10 000 live births and characterized by malformations of the peripheral nervous system, craniofacial structures, eyes, ears, and heart.^26^ The role of CHD7 in development has been widely studied, but its role in tumourigenesis is poorly understood. In our pan‐cancer multi‐omics analysis of ATPCRs, *CHD7* is one of the genes with a high comprehensive score: *CHD7* had a high frequency of somatic mutations and increased copy number in a variety of cancers; *CHD7* was associated with more than 10 transcript fusion events in tumours; compared with normal tissues, *CHD7* was highly expressed in a variety of tumour tissues; therefore, it was presumed that *CHD7* plays a key role in tumourigenesis. As *CHD7* had the highest score in CRC, we conducted experimental verification in CRC cells to further verify our analyses. The results of a series of cellular experiments and mouse xenograft experiments demonstrated that CHD7 promotes the growth of CRC in vitro and in vivo. The oncogenic role of CHD7 in lung adenocarcinoma was also verified (Figure S5). As *CHD7* is often mutant in CHARGE syndrome, we compared the locations of the mutations identified in CRC and CHARGE syndrome. Our analyses showed 55 mutations in CRC which are dominant of missense mutations, and 53 mutations in CHARGE syndrome which are dominant of truncating mutations, and there was no overlapped site (Figure S6), indicating that the mutations in *CHD7* that drive CRC and CHARGE syndrome are totally different.

CHD7 has been reported to catalyse nucleosome movement on DNA to affect chromatin accessibility.^6^ We then investigated the effect of CHD7 knockdown on genomic accessibility through an ATAC‐seq experiment. In our results, we found that knockdown of CHD7 resulted in changes in chromatin accessibility in many regions of the genome. After knockdown of CHD7, the regions with enhanced chromatin accessibility (cluster 1) were mainly concentrated in the distal intergenic (62.76%), which may function as distal enhancers. However, the regions (cluster 2) with decreased chromatin accessibility after CHD7 knockdown were mainly concentrated in the promoters (66.94%). Combined with the results of RNA‐seq, ATAC‐seq, and ChIP experiments, we showed that CHD7 could bind to the promoter region of target genes (such as *AK4*, *UTP14A*, and *TFAP4*) to maintain chromatin accessibility and facilitate transcription. AK4, an adenylate kinase, can regulate cellular ATP levels and AMPK signalling. Knockdown of AK4 leads to an increase in AMP/ATP level and the activation of AMPK.^19^ Although it has been reported that knockdown of AK4 can significantly inhibit the migration and invasion of CRC cells under hypoxia,^27^ the specific molecular mechanism and its effect on tumour cell proliferation are still unknown. In our study, we found that AK4 and CHD7 knockdown increased AMPK phosphorylation levels in CRC cells. AMPK activation has been reported to catalyse the phosphorylation of p53.^20^ We further demonstrated that knockdown of AK4 and CHD7 increased the phosphorylation level of p53 at S15, S46, and S492 sites, which was reported to improve the protein stability and transcriptional activity of p53. Moreover, AK4 overexpression rescued the inhibited CRC cell growth and the increased p53 protein expression induced by CHD7 knockdown, demonstrating that CHD7 exerted its oncogenic role through AK4. Our work reported for the first time that downregulation of AK4 can increase the protein stability and activity of p53 and explored a novel CHD7‐AK4‐AMPK‐p53 axis that plays an oncogenic role in CRC, providing several potential therapeutic targets of CRC.

## 4 MATERIALS AND METHODS

### 4.1 Analysis of SCNA data and ATPCRs driven by recurrent SCNAs

SCNA data were downloaded from the Genomic Data Commons Data Portal (https://portal.gdc.cancer.gov/), and the CNV segmentation files were used as inputs for the GISTIC 2.0 algorithm^15^ (https://www.broadinstitute.org/cancer/cga/gistic) to identify genes targeted by SCNAs that may drive cancer growth. We set the confidence level of GISTIC to .99, and ATPCRs located in the peak region of gains or losses were considered to be significantly amplified or deleted. For a specific cancer type, we adopted two criteria to obtain the ATPCRs that were significantly targeted by SCNAs: (1) located in peak regions of recurrent SCNAs defined by GISTIC (*q* ≤ 0.25) and (2) altered with a high frequency and large amplitude (*G*‐score ≥ 0.1). Using an unweighted summation of *G*‐scores from all cancer types, we calculated a pan‐cancer *G*‐score at a pan‐cancer level to estimate SCNAs for potential cancer driver ATPCRs.

### 4.2 Significantly mutated gene analysis

Whole‐exome sequencing data of 32 cancer types were acquired from the TCGA MC3 project^28^ (https://doi.org/10.7303/syn7214402), which is an integrated result from seven independent mutation calling algorithms as follows: Pindel^29^ (INDEL), MuSE^30^ (SNV), Radia^31^ (SNV), VarScan2^32^ (SNV/INDEL), MuTect^33^ (SNV), Indelocator^34^ (INDEL), and SomaticSniper^35^ (SNV). Additionally, a collection of filtering methods was performed. The mutation frequency of each gene was calculated by dividing the number of mutated specimens by the total number of specimens. Furthermore, we selected five previously published complementary methods for screening potential cancer‐related ATPCRs that are driven by mutations: MutSigCV^36^ (http://software.broadinstitute.org/cancer/software/genepattern/modules/docs/MutSigCV), OncodriveCLUST^37^ (https://bitbucket.org/bbglab/oncodriveclustl), ActiveDriver^38^ (http://reimandlab.org/software/activedriver/), iPAC^39^ (http://www.bioconductor.org/packages/2.12/bioc/html/iPAC.html), and DriverML^40^ (https://github.com/HelloYiHan/DriverML). We used MC3 MAF files as inputs for the previously mentioned five algorithms and used the default parameters. We set the *m*‐index (0–5) indicating the number of previous five algorithms that contributed to identifying a driver gene. Genes with an *m*‐index ≥2 were regarded as significantly mutated.

### 4.3 Transcript fusion data analysis

We downloaded the transcript fusion data of TCGA from the TumorFusions data portal (http://tumorfusions.org/), which contained 20 731 gene fusion events detected in 9950 well‐characterized cancer specimens and 648 normal specimens across 32 cancer types from TCGA.^16^ A uniform pipeline was used to identify fusion transcripts in RNA‐seq data analysis^41^ using a positive control fusion list, including resources from Mitelman, ChimerPub, and Cosmic fusions. Recurrent gene fusions were defined as occurring at least twice across all cancer specimens. The Circos plot of transcript fusions was drawn using the ‘Circos’ package in R.

### 4.4 RNA‐seq data analysis

Normalized gene expression data (FPKM data) from TCGA were obtained from the UCSC Xena browser (http://xena.ucsc.edu/), which incorporated 32 cancer types.^42^ As some cancers do not have data from paracancerous tissues, we compared gene expression levels between tumour specimens and normal adjacent specimens for 22 cancer types by performing gene differential expression analysis based on the Wilcoxon signed‐rank test.

### 4.5 Copy number alterations and RNA expression correlation analysis

To clarify the correlation between RNA expression and copy number of ATPCRs, we selected genes with an FPKM ≥ 1 in at least 90% tumour specimens of a certain cancer type and then correlated the expression data with copy number values from GISTIC results. A Pearson correlation test was used to determine whether the correlation was positive with a *p*‐value cut‐off of 0.001.

### 4.6 Survival data analysis

The clinical data of 32 cancer types were obtained from the UCSC Xena browser (http://xena.ucsc.edu/).^42^ We focused on the OS status to evaluate whether the expression of ATPCRs affected patient prognosis. Each gene was used as a univariate factor to the survival formula of the ‘Survival’ package in R, and results with *p* < 0.1 and HR > 1 were considered to be poor prognosis, whereas *p* < 0.1 and HR < 1 were considered a good prognosis.

### 4.7 CS‐ and PCO‐score calculation

Combined with the genomic aberrations, transcriptome, and clinical outcome data, we determined a CS‐score for each ATPCR in each cancer type to evaluate its cancer driver potential. In a specific cancer type, we assigned an integer number (ranging from 0 to 5) to the CS‐score of ATPCRs, which indicates how many of them satisfied the following conditions: (1) recurrent focal SCNAs, (2) significantly mutated, (3) recurrent fusions, (4) significantly different expression in cancer tissues in contrast to the normal tissues; and (5) prediction of good or poor prognosis. The PCO‐score was obtained by summing over the CS‐scores of all cancer types. To be specific, CS‐ and PCO‐score of each ATPCR were calculated using the following formulas^43^:

$$\begin{array}{ccc} \text{CS-score} & = & \Sigma_{k=1}^{5}\;X,\;X\in \left\{0,1\right\}\\ \text{PCO-score} & = & \Sigma_{n=1}^{i}\;\Sigma_{k=1}^{5}\;X,\;X\in \left\{0,1\right\} \end{array}$$

where *k* represents the number of the previously mentioned five conditions for a certain cancer type, and *i* represents the number of cancer types.

ATPCRs with high CS‐ and PCO‐score were more likely to be putative cancer driver genes.

### 4.8 Cells and reagents

HCT116 and RKO cells were cultured in Dulbecco's Modified Eagle's Medium (DMEM) or Eagle's Minimum Essential Medium supplemented with 10% foetal bovine serum (Biological Industries, Beit HaEmek, ISRAEL) at 37°C in a humidified atmosphere with 5% CO_2_. Cell lines were authenticated by examining their morphology and growth characteristics. Anti‐CDKN1A (A5952), anti‐β‐actin (AC038), anti‐TP53INP1 (A5952), anti‐p‐p53‐S46 (AP046), anti‐p‐p53‐S392 (AP0860), anti‐p‐p53‐S376 (AP0987), anti‐p‐p53‐S15 (AP0950), anti‐AMPKα (A12718), anti‐p‐AMPK (AP0116), and anti‐AK4 (19854) were purchased from ABclonal Technology Co. (Wuhan, Hubei, CN). Anti‐CHD7 (A301‐223A) were purchased from Bethyl Laboratories Inc. (Montgomery, AL, USA). Anti‐Ki67 (D2H10) was purchased from Cell Signaling Technology (Danvers, MA, USA). Anti‐FLAG (M2, F3165) and anti‐α‐tubulin (SAB4500087) were purchased from Merck KGaA (Darmstadt, Germany). Anti‐TP53 (SC‐126) antibodies were purchased from Santa Cruz Biotechnology (Santa Cruz, CA, USA). Fluorescein (111‐095‐003 and 115‐095‐003) secondary antibodies were obtained from Jackson ImmunoResearch Laboratories (West Grove, PA, USA) and horseradish peroxidase‐conjugated secondary antibodies (sc‐2030 and sc‐2031) were purchased from Santa Cruz Biotechnology. 4‐6‐Diamidino‐2‐phenylindole was purchased from Merck KGaA.

### 4.9 Soft agar assay

Cells (3000 cells/well) were suspended in .35% agar prepared in DMEM containing 10% FBS and plated onto a layer of .7% agar in DMEM supplemented with 10% FBS in 6‐well plates. After 2 weeks, the colonies were stained with MTT solution (MTT final Con. .5 mg/ml) at 37°C for 4 h and then photographed.

### 4.10 Subcutaneous xenograft model

Lentiviruses expressing *CHD7* shRNAs or control shRNAs were used to infect RKO cells, which were then subcutaneously injected into the right flanks of nude mice (BALB/c, Charles River; 5–6 weeks of age; six mice per group). Tumour volume was measured every 3 days, and the volume was calculated. The mice were sacrificed 15 days post‐injection. Tumours were then stripped out and photographed. A specific time point was not selected to assess significance. Animal handling and procedures were approved by the Institutional Animal Care and Use Committees of Tianjin Medical University.

### 4.11 ATAC‐seq

ATAC‐seq was performed as previously described. In brief, 100 000 cell nuclei were extracted using NPB buffer (10‐mM Tris, pH 7.5, 10‐mM NaCl, 3‐mM MgCl_2_, and .1% Triton X‐100) at 4°C for 10 min. Tagmentation was performed in a reaction buffer (Vazyme) containing Tn5 transposase (#TD501, Vazyme, Nanjing, China) at 37°C for 30 min. DNA was then extracted using a PCR purification kit (#28106, Qiagen, Hilden, Germany), and DNA libraries were generated by PCR. The PCR products were purified using Ampure beads (#12601ES03, Yeason, Shanghai, China) and subjected to deep sequencing (Novogene, Beijing, China).

### 4.12 ChIP (chromatin immunoprecipitation)

Cells were washed twice with PBS, crosslinked with 15‐ml 1% formaldehyde solution for 10 min at room temperature and then quenched with 1 ml of 2‐M glycine (final concentration, .125 M). After rinsing twice with PBS, cells were harvested in SDS buffer (50‐mM Tris–HCl, pH 8.0, 100‐mM NaCl, 5‐mM EDTA, and 10% SDS) containing protease inhibitors. Cell pellets were collected by spinning for 6 min at 1200 rpm and resuspended in ice‐cold IP buffer (100‐mM NaCl, 66.67‐mM Tris–HCl, pH 8.0, 5‐mM EDTA, .33% SDS, and 1.67% Triton X‐100) followed by sonication using Bioruptor (Diagenode, Liege, Belgium). After centrifugation at 13 000 rpm for 15 min at 4°C, CHD7 antibodies were added to the supernatant for rotation overnight in a cold room. Protein A/G beads were then added for another 3 h at 4°C. Beads were washed sequentially with wash buffer 1 (150‐mM NaCl, .1% SDS, 1% Triton X‐100, 2‐mM EDTA, and 20‐mM Tris‐–HCl, pH 8.0) three times, and wash buffer 2 (1% Triton X‐100, 500‐mM NaCl, .1% SDS, 2‐mM EDTA, and 20‐mM Tris–HCl, pH 8.0) once, followed by reverse crosslinking for 5 h at 65°C. DNA was extracted and subjected to quantitative PCR using primers targeting the promoters of target genes.

### 4.13 Immunohistochemistry analysis

Briefly, transplanted tumours dissected from mice were infiltrated with O.C.T. compound (#4583, SAKURA, CA, USA) and solidified at −20°C. The embedded specimens were cut into 8‐μm serial sections and stored at −80°C. The sections were treated with 3% hydrogen peroxide in the dark for 15 min to quench endogenous peroxidases and then blocked in PBST (PBS with .5% Triton‐X100) containing 10% goat serum for 2 h. Primary antibodies were diluted in 10% goat serum in PBST and incubated with the sections overnight at 4°C. Sections were washed three times with PBST and incubated with a biotinylated secondary antibody at RT for another 2 h. Sections were then incubated with DAB substrate solution for 5–10 s and counterstained with hematoxylin. Finally, sections were dehydrated and mounted on coverslips.

### 4.14 Statistical analysis

Statistical analysis was performed using R software (v3.6.3). All results are presented as mean ± SD, and *P* < 0.05 (except survival analysis with *P* < 0.1) indicated statistical significance.

## CONFLICT OF INTERESTS

The authors declare that there is no conflict of interest that could be perceived as prejudicing the impartiality of the research reported.

## Supporting information

Figure S1 Summary of the genomic alteration events of ATPCRs across cancer types
Figure S2 Summary of the expression of ATPCRs across cancer types.
Figure S3 Summary of the CNVs of CHD7 in CRC
Figure S4 CHD7 depletion does not affect the growth of p53‐mutant CRC cells in vitro.
Figure S5 CHD7 promotes the growth of lung adenocarcinoma cells in vitro.
Figure S6 Variant sites of CHD7 in CRC and CHARGE syndrome

Supplement Material

Table S1 Summary of TCGA specimens

Table S2 Summary of the ATPCRs located in the recurrent focal SCNAs identified

Table S3 Pan‐cancer level *G*‐score of ATPCRs that recurrently gained or lost in at least

Table S4 List of the driver ATPCR genes in each cancer type by somatic mutation

Table S5 Summary of mutation frequency of the ATPCRs in each cancer type

Table S6 List of the transcript fusions of the ATPCRs in each cancer type

Table S7 Summary of ATPCRs expression percentage in each cancer type

Table S8 Summary of differently expressed ATPCR genes in each cancer type

Table S9 Summary of ATPCRs significant correlation between expression and CNVs in different cancer types

Table S10 Summary of the overall survival of the ATPCRs across all

Table S11 Summary of the PCO‐score of the ATPCRs across all cancer

Table S12 Summary of the CS‐score of the ATPCRs in a given cancer type

## Data Availability

The raw data, processed data, and clinical data used for the study are available through the Genomic Data Commons (GDC) portal (https://gdc‐portal.nci.nih.gov), the UCSC Xena browser (http://xena.ucsc.edu/), the TCGA MC3 files (https://doi.org/10.7303/syn7214402), and TumorFusions data portal (http://tumorfusions.org/). The raw and processed high‐throughput RNA‐seq and ATAC‐seq data were deposited in the Gene Expression Omnibus (GEO) database under accession number GSE192725 (https://www.ncbi.nlm.nih.gov/geo/query/acc.cgi?acc=GSE192725) and GSE192905 (https://www.ncbi.nlm.nih.gov/geo/query/acc.cgi?acc=GSE192905), respectively.
