# Supplementary material for: Integrated analysis of genes encoding ATP‐dependent chromatin remodellers identifies CHD7 as a potential target for colorectal cancer therapy
Source: Clin Transl Med. 2022 Jul 5;12(7):e953. doi: 10.1002/ctm2.953 (PMC9254903; doi:10.1002/ctm2.953)
Supplement: Supplementary file 1 — Figure S1 Summary of the genomic alteration events of ATPCRs across cancer types Figure S2 Summary of the expression of ATPCRs across cancer types. Figure S3 Summary of the CNVs of CHD7 in CRC Figure S4 CHD7 depletion does not affect the growth of p53‐mutant CRC cells in vitro. Figure S5 CHD7 promotes the growth of lung adenocarcinoma cells in vitro. Figure S6 Variant sites of CHD7 in CRC and CHARGE syndrome [file CTM2-12-e953-s006.pdf]

Supplementary Figures

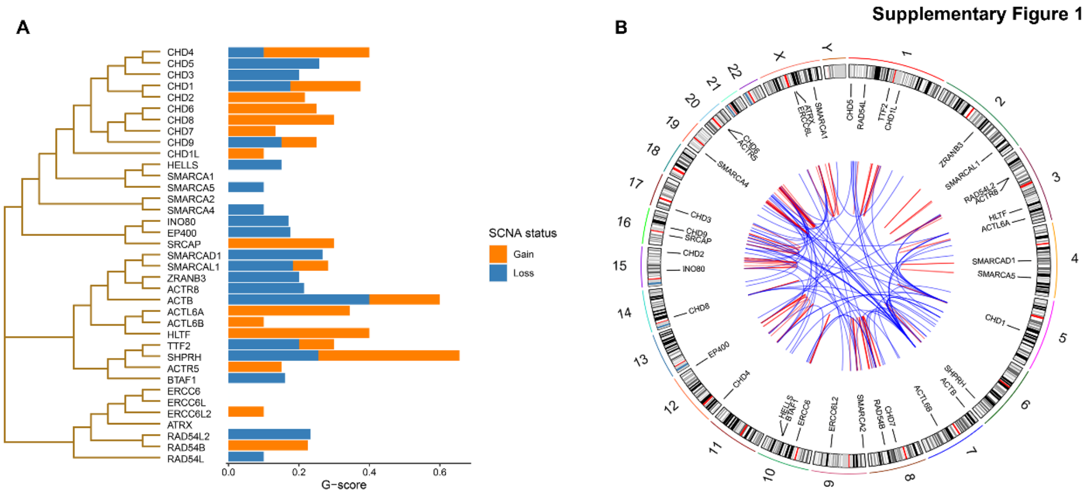

Supplementary Figure 1. Summary of the genomic alteration events of ATPCRs across cancer types. (A) Summary of the pan-cancer G-scores of ATPCRs driven by SCNAs. The length of the bars represents the pan-cancer G-score. The phylogenetic trees were generated by maximum likelihood methods based on protein sequences of ATPCRs. **(B)** The Circos plot shows the recurrent fusions involved in ATPCRs. The fusion genes are linked by lines. Red lines denote intra-chromosome fusions and blue lines denote inter-chromosome fusions.

Supplementary Figure 2

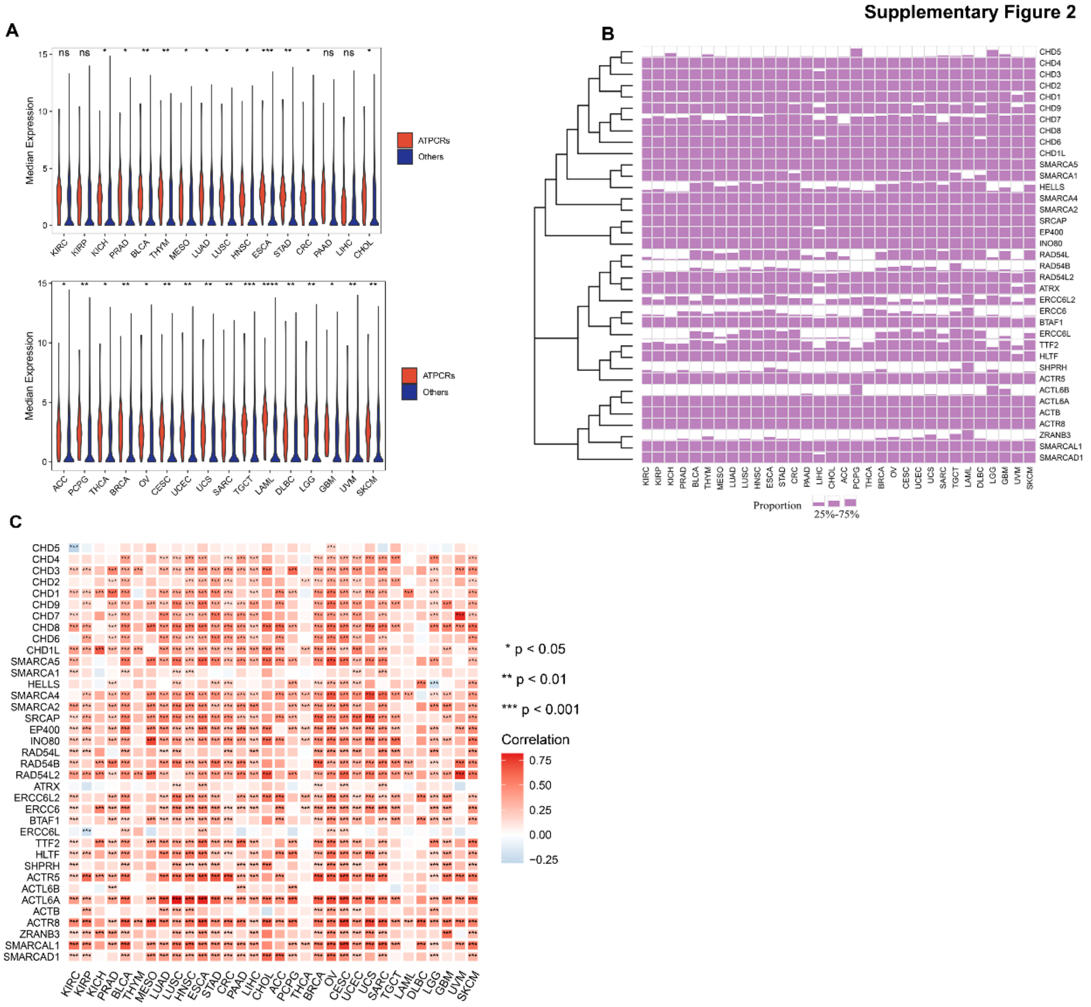

Supplementary Figure 2. Summary of the expression of ATPCRs across cancer types. (A) The violin plot shows the comparison of the median expression values of the ATPCRs with other coding genes in different cancer types. *P < 0.05, **P < 0.01, ***P < 0.001 (Wilcox test). **(B)** Expression of ATPCRs across cancer types. The heat map shows the mRNA expression rate of ATPCRs across cancer types. The purple part of the cells represents the percentage of specimens that express certain ATPCRs in certain cancer types, with a filtration threshold of per kilobase million (FPKM) ≥ 1. **(C)** The heatmap shows the correlation coefficient between copy number and expression of ATPCRs. The colors represent the correlation coefficient and the asterisks represent the significance.

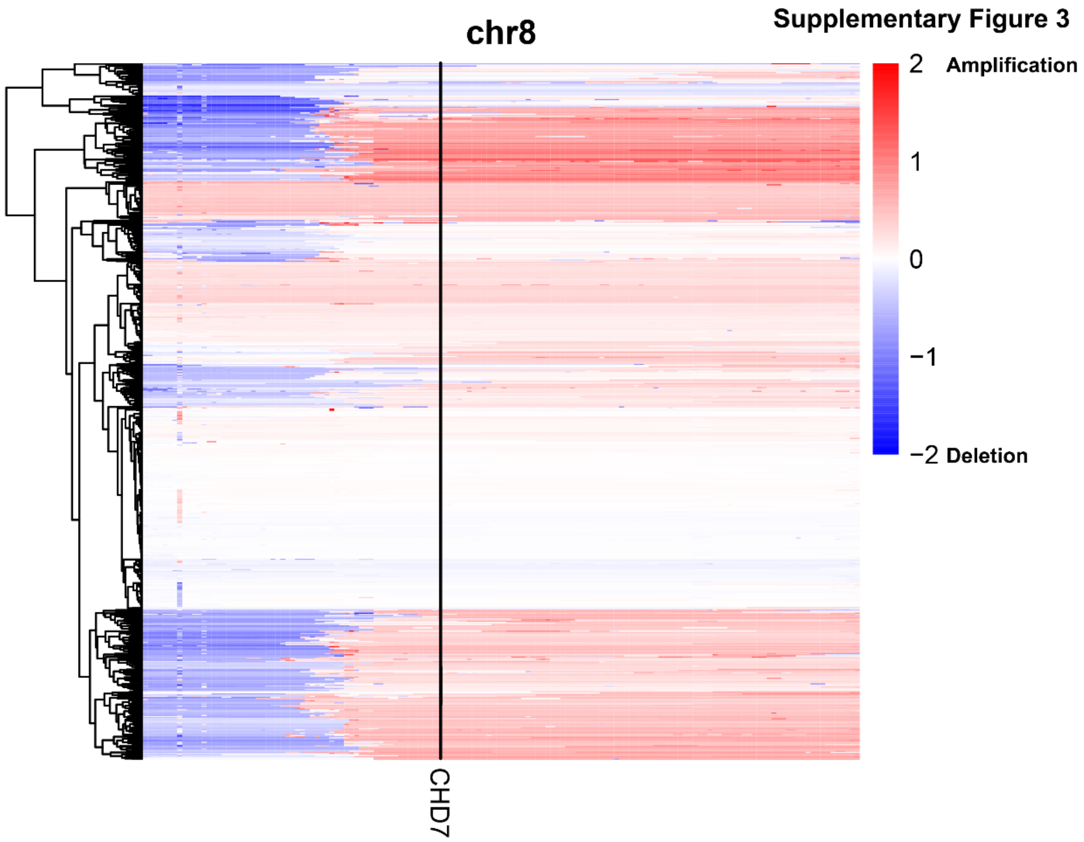

Supplementary Figure 3. Summary of the CNVs of CHD7 in CRC. The heatmap shows the copy number changes on chromosome 8 in TCGA CRC cohort. The colors of blue and red represent deletion and amplification, respectively. The location of CHD7 was marked as a black line.

Supplementary Figure 4

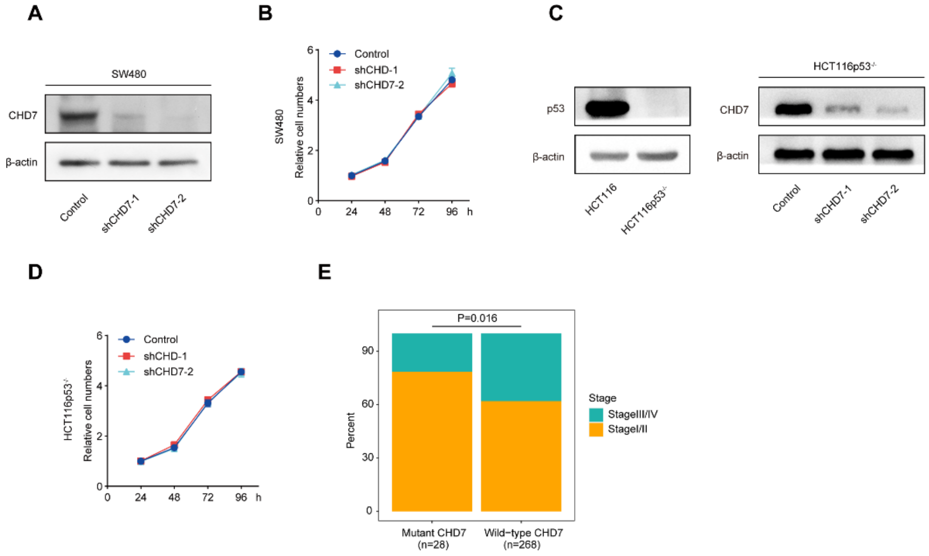

Supplementary Figure 4. CHD7 depletion doesn't affect the growth of p53-mutant colorectal cancer cells *in vitro*. (A) CHD7 was knocked down by two independent shRNAs in SW480 cells. Knockdown efficiency was detected by Western blotting. **(B)** Depletion of CHD7 doesn't affect the viability of SW480 cells. Cell viability was measured using MTT assays. **(C)** The knockdown efficiency of CHD7 shRNAs in HCT116 $p53^{-/-}$ cells, and the expression of p53 in HCT116 and HCT116 $p53^{-/-}$ cells was detected by Western blotting. **(D)** Depletion of CHD7 doesn't affect the viability of HCT116 $p53^{-/-}$ cells examined by MTT assays. For figures B and D, Data are mean ± SD for n = 3. **(E)** The bar chart shows the percentage of late-stage and early-stage cases in mutant CHD7 and wild-type CHD7 groups of colorectal cancer patients with wild-type p53. The *P* value (Student's t test) was marked on the top of the chart.

Supplementary Figure 5

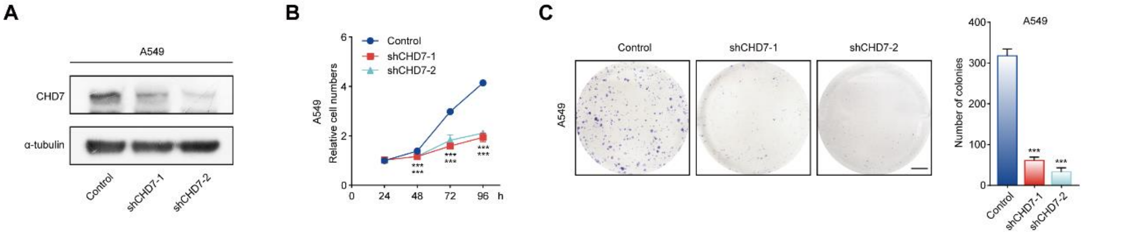

Supplementary Figure 5. CHD7 promotes the growth of lung adenocarcinoma cells *in vitro*. (A) CHD7 was knocked down by two independent shRNAs in A549 cells. Knockdown efficiency was detected by Western blotting. **(B)** Depletion of CHD7 decreases the viability of lung adenocarcinoma cells. Cell viability was measured using MTT assays. **(C)** A549 cells expressing indicated shRNAs were maintained in culture media for 2 weeks and stained with crystal violet, and the number of colonies was counted. Scale bar, 5 mm. Data are mean ± SD for n = 3; ***P < 0.001 (Student's t test).

Supplementary Figure 6

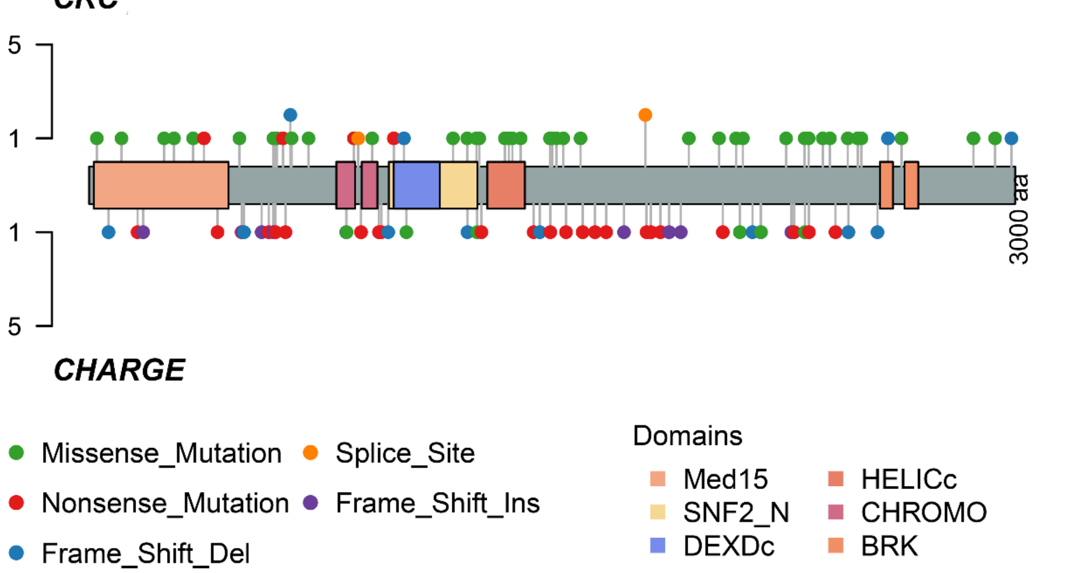

Supplementary Figure 6. Variant sites of CHD7 in CRC and CHARGE syndrome. The lollipop plot shows mutations of CHD7 in CRC and CHARGE syndrome. The different colors of the balls on the stick chart represent different types of mutations. The different colors of blocks represent different domains of CHD7.
