# Supplementary material for: Integrated analysis of genes encoding ATP‐dependent chromatin remodellers identifies CHD7 as a potential target for colorectal cancer therapy
Source: Clin Transl Med. 2022 Jul 5;12(7):e953. doi: 10.1002/ctm2.953 (PMC9254903; doi:10.1002/ctm2.953)
Supplement: Supplementary file 2 — Supplement Material [file CTM2-12-e953-s008.pdf]

## Supplementary Materials and Methods

### MTT assay

Cell viability was detected using the 3-(4, 5-dimethylthiazol-2-yl)-2, 5-diphenyl-2H-tetrazolium bromide (MTT) assay. In brief, 1,500 cells suspended in 100 µl DMEM with 10% FBS were seeded into a well of a 96-well plate and incubated for 24, 48, 72, or 96 hours. Then, medium was replaced with MTT solution prepared in DMEM (MTT final Concentration is 0.5 mg/ml). After incubation for 4 hours at 37 °C, the medium was discarded and cells were lysed for 30 minutes with DMSO at room temperature. The absorbance was spectrophotometrically measured at 490 nm.

### Apoptosis assay

Apoptosis was examined using the FITC Annexin V Apoptosis Detection Kit (AO2001-02P-G, Sungene Biotech, Tianjin, China) according to the manufacturer's instructions. In brief, cells were digested with trypsin-EDTA into a single-cell suspension and collected by centrifugation at 200 × g for five minutes. The cells were resuspended in 100 µl of Annexin V binding solution containing 5 µl of annexin V-FITC. After incubation for 10 minutes at room temperature in the dark, 5 µl of propidium iodide solution was added to the tube and incubated for 5 minutes. Then, 400 µl of PBS was added to the cell suspension which was then subjected to a flow cytometer (Accuri C6, BD Biosciences, Franklin Lakes, NJ, USA).

### EdU-incorporation assay

EdU (5-ethynyl-2’-deoxyuridine)-incorporation assays were performed according to the manufacturer’s instructions (C0078S, Beyotime, Beijing, China). Briefly, cells were cultured in wells of a 96-well plate for 24 hours. The culture medium was then replaced with medium containing EdU for 2 hours. Cells were fixed, permeabilized, stained with Hoechst 33342, and observed under a fluorescence microscope.

**Colony formation assay**

One thousand cells were plated in 6-well plates. After 2 weeks’ culture, cells were washed twice with PBS and fixed for 15 minutes in 4% paraformaldehyde. Colonies were then stained with 0.1% crystal violet staining solution for 10 minutes, and photographed.
